# Supplementary material for: Population genomics of C. melanopterus using target gene capture data: demographic inferences and conservation perspectives
Source: Sci Rep. 2016 Sep 21;6:33753. doi: 10.1038/srep33753 (PMC5030670; doi:10.1038/srep33753)

Supplementary Information for the manuscript titled:

"Population genomics of *C. melanopterus* using target gene capture data: demographic inferences and conservation perspectives"

Pierpaolo Maisano Delser[1,2,¶], Shannon Corrigan[3,¶], Matthew Hale[4], Chenhong Li[3,5], Michel Veuille[1,2], Serge Planes[6], Gavin Naylor[3,&], and Stefano Mona[1,2,&,*]

[1] [1] Institut de Systématique, Évolution, Biodiversité, ISYEB - UMR 7205 - CNRS, MNHN, UPMC, EPHE, Ecole Pratique des Hautes Etudes, 16 rue Buffon, CP39, 75005, Paris, France

[2] EPHE, PSL Research University, Paris, France

[3] Department of Biology, College of Charleston, Charleston 29412, SC, USA

[4] Medical University of South Carolina, College of Graduate Studies, Charleston 29403, SC, USA

[5] Key Laboratory of Exploration and Utilization of Aquatic Genetic Resources, Shanghai Ocean University, Ministry of Education, Shanghai 201306, China

[6] CRIOBE-USR 3278, CNRS-EPHE-UPVD, Laboratoire d'Excellence 'CORAIL', 58 Avenue Paul Alduy, 66860 Perpignan, France.

[*] mona@mnhn.fr (SM)

[¶] These authors contributed equally to this work

[&] These authors contributed equally to this work

Materials and Methods

Bait design for target enrichment

A custom biotinylated RNA bait library (MYbaits MYcroarray® Ann Arbor, MI, USA), based on sequences derived from seven species (*Chlamydoselachus anguineus, Etmopterus joungi, Isurus oxyrinchus, Orectolobus halei, Carcharhinus amblyrhynchos, Heterodontus portusjacksoni,* and *Squatina nebulosa*) spanning the ordinal diversity of sharks, was used to target corresponding putative orthologs in the target libraries of each sample of *C. melanopterus*[1] .

Target library preparation and sequencing

A single set of universal primers[2] and Polymerase Chain Reaction (PCR) were first used to amplify the mitochondrial DNA NADH2 fragment for all samples. This fragment is particularly useful for distinguishing elasmobranch species[3] and resulting sequences were compared against a database containing more than 12,000 elasmobranch NADH2 sequences[3] in order to confirm the species identification of each sample prior to further analysis.
We sequenced 1077 independent autosomal regions using target gene capture[1]. Genomic DNA for each *C. melanopterus* sample was first sheared to approximately 500bp using acoustic ultrasonication to form a target DNA library. Illumina sequencing adapters were ligated to the sheared fragments. Target libraries were amplified by PCR prior to two rounds of target gene capture using a 'touchdown' DNA hybridization approach[1]. The biotinylated bait-target complex resulting from capture was retrieved by binding to streptavidin beads and washing away unbound and weakly bound non-target DNA. The resulting library was now enriched for on-target material and was re-amplified to incorporate a sample specific index prior to sequencing. All samples were pooled in equimolar ratios and the pooled library was diluted to 12 pM for paired-end 250 bp sequencing on an Illumina MiSeq benchtop sequencer (Illumina, Inc, San Diego, CA). Sequence reads associated with each sample were identified and sorted by their respective indices.

Reference Sequence

Sequence read data from several individuals was used to build a reference sequence for the 1077 target exons and associated introns. Adapters were trimmed from sequence reads and low quality reads removed using cutadapt and FastQC available in the wrapper script Trim Galore! v0.3.1[4]. *De novo* assembly of trimmed sequence reads was performed in ABySS v1.3.6[5] using multiple k-mer values ranging, in increments of 10, between k = 51 and 251. Assembled contigs were filtered, extended and merged using Trans-ABySS v.1.4.4[6]. HaMStR v9[7] was used to assign each assembled contig to one of the 1,077 core ortholog groups. HaMStR v9 uses a combination of BLASTP[8], GeneWise[9], and HMMER[10], to search the target database of assembled contigs for protein sequences that match a core ortholog set. The core ortholog database consisted of 1077 profile hidden Markov models (pHMMs)[11] that characterise orthologous sequence groups obtained from six model vertebrate genomes: *Anolis carolinensis, Callorhinchus milii, Danio rerio, Gallus gallus, Homo sapiens* and *Xenopus tropicalis*. Any contig that matched one of the core-ortholog pHMMs with a default E-value less than 1.0 was initially regarded as a "hit" and was provisionally assigned to that orthologous group. Hits against the pHMMs were then compared to the "reference taxon",

Callorhinchus milii (a Chondrichthyan fish and therefore the closest relative to *C. melanopterus* of the available model vertebrates), and retained only if they fulfilled a reciprocal best BLAST hit criterion with that taxon. When multiple contigs fulfilled the orthology criteria for a particular locus, the sequence with the best pairwise alignment to the reference taxon, *C. milii,* was chosen as the representative for that locus (-REPRESENTATIVE option in HaMStR v9). The resulting 1077 putative orthologous protein sequences were back translated to nucleotide sequences. Although our RNA baits target coding DNA regions, flanking intron regions are also captured and sequenced. Searching for orthologous nucleotide sequences identified by HaMStR within original assembled contig strings allowed us to retrieve flanking 5′ and 3′ intron sequence. This was achieved using a custom Perl script. For each exon and intron (5′ and 3′) the longest contig across all sequenced individuals was retained and used to assemble the reference sequence. Positions carrying gaps across all individuals were discarded to avoid missing data in the final reference sequence. Gap positions in the longest contig were substituted with information from other samples where possible. Because these fragments are not contiguous, 200 bp of Ns were added at the beginning and end of each contig to facilitate alignment. The length of the final concatenated reference sequence is 1,293,710 bp including exons and introns (5′ and 3′), and 1,724,510 bp when Ns are also considered.

Data analysis, variant calling and filtering

Quality control checks were performed with FastQC (http://www.bioinformatics.bbsrc.ac.uk/projects/fastqc/) on the raw fastq files before and after adapter removal which was done with fastq-mcf[12]. Reads were mapped to the reference sequence using a Burrows-Wheeler Aligner (BWA v0.7.10)[13]. Duplicate read marking was done with Picard v1.129 (http://broadinstitute.github.io/picard/) followed by local realignment performed with The Genome Analysis Toolkit (GATK) v3.3-0[14]. The individual steps and parameters used are listed in Supplementary Table 8.

The samtools mpileup v1.1[14] multi-sample option was used for variant calling, treating all samples simultaneously with the following general parameters: minimum base quality (BQ) 20, minimum mapping quality (MQ) 30 and no INDEL calling (Supplementary Table S8). In total 1,268,572 sites were called out of which 9315 were SNPs.

Raw variants were filtered using custom Perl and R scripts (Supplementary Table S8, S5). Filtering based on minimum depth (DP≥6) and strand bias (SP≤13) was carried out at the genotype level. Then samples were divided in two datasets called "scatter" (SCD) and "single deme" (SID) with 9 and 11 samples respectively (Supplementary Table S1). At this stage, any site carrying a genotype that was filtered out in the previous steps was discarded in order to have a dataset without any missing data (NA=0). Loci were discarded because either no site passed the filters or a pattern compatible with duplicated elements was present. In the latter case, these regions were manually investigated and then eventually discarded to avoid the introduction of an artificial excess of heterozygous sites generated by alignment of multiple copies of a duplicated element to the reference sequence. This pattern affected on average 10% of the discarded loci between SCD and SID and these regions were consistent across the two datasets. Triallelic sites were also discarded and the SNP density for each region was calculated. Regions with an excess of SNPs were manually investigated and discarded if considered dubious (i.e. structural variation). The average length of the regions is ~600bp and details of the length distribution for SCD and SID are shown in Supplementary Figure 1.

Mean raw sequence coverage per sample was calculated using GATK v3.3-0[14]. Sequence depth for the 18 samples varied from 46× to 136× per sample, with the average of 75×.

A summary for the filtering steps and details for both datasets are shown in Supplementary Table 2.

Sequencing error rate

The sequencing error rate was assessed by comparing two replicates of the same individual. All laboratory procedures from DNA extraction through library preparation and gene capture were conducted independently for each of the replicates. Both replicates were sequenced as part of one pooled library in a single MiSeq run. Comparison was based on 741,328 bp and 104 differences were found leading to an estimate of the sequencing error rate of 0.014%.

Calibration of the mutation rate

We used the appearance of the isthmus of Panama at 3 MYA[15] to calibrate the molecular clock in the absence of fossil records. We collected six samples of *Carcharhinus galapagensis* from 3 different locations grouped in either the Atlantic or Pacific side of the isthmus of Panama: Bermuda (N=3) from the Atlantic side, Galapagos (N=2) and Hawaii (N=1) for the Pacific side. *C. galapagensis* samples were typed using the same target gene capture approach as in *C. melanopterus* in order to obtain an average mutation rate along the same genomic regions used for our demographic inferences.

Analysis was performed with BEAST[16] using a Yule tree prior with a uniform prior distribution for the clock rate between 10^{-10} and 10^{-8}, a normal prior distribution for the tree root height (calibration point, the appearance of the isthmus of Panama) with mean 3 million years [15] and standard deviation 10,000 years and HKY + gamma as the substitution model[17]. We ran the dataset for 10,000,000 iterations with a 10% burn-in and a thinning of 1000. Convergence was checked by visualising and examining the traces using TRACER v 1.6[18]. The 95% high posterior density of the mutation rate ranged between 1.15 and 1.22×10^{-9} nucleotide/year. Considering 7 years as the generation time for *Carcharhinus melanopterus*[19], we obtained an estimation of the mutation rate ranging between 8.05 and 8.54×10^{-9} nucleotide/generation.

Principal Component Analysis

A Principal Component Analysis (PCA) was performed on the whole dataset (18 samples) with the function "prcomp" in the R environment[20]. The first principal component separates the Australian samples from the rest of the dataset while the second principal component isolates Oman (Supplemenatry Fig. 2, panel a and b). Overall, the first two principal components explain ~40% of the variance. To further investigate the level of population structure in our dataset, we removed the Oman samples repeated the PCA. The first principal component still divides the Australian samples from the rest of the dataset while the second principal component highlights patterns of population substructure within both the Australian continent (Queensland and Northern Territory) and Indonesia (Supplementary Fig. 2, panel c and d). Overall, we can exclude the presence of a panmictic population. The genetic distances between populations were also assessed by computing a Gst matrix[21] (Supplementary Table 5) which suggests equal distances among populations with the exception of the samples coming from Oman.

Approximate Bayesian Computation approach

We developed an ABC[22] framework to estimate parameters and compare models. In our ABC approach, we simulated exactly the same number and length of the observed regions for each dataset (995 and 998 regions for SCD and SID respectively). In this way, we built our simulation to have the same configuration as the observed data, also accounting for differences in the length of the regions. We let mutation and recombination rates vary across loci by setting a normal hyper prior distribution on both of them. The mean of the hyperprior distribution of the mutation rates was modelled as uniform bounded between 8.05 and 8.54 x 10^{-9} per site per generation, following the calibration for *C. galapagensis*. Having no prior information on the recombination rate of species close to *C. melanopterus*, a uniform distribution between 0 and 10^{-8} was chosen for the mean of the hyper prior distribution on the recombination rate. For the standard deviation on both the hyper prior distributions on mutation and recombination rate, a uniform distribution between 10^{-11} and 10^{-10} was applied. Such hyperprior distributions on mutation and recombination rates allowed us to account for variation in both mutation across the genome. Moreover, by modelling intra-locus recombination we could use multiple SNPs coming from the same region. We selected the folded site frequency spectrum (SFS) and the total number of SNPs as summary statistics to avoid phasing issues. Four demographic models were tested for both datasets (Fig. 2).

We generated 100,000 simulations for each demographic model using fastsimcoal2 version 2.5.1[23]. Each simulation includes 995 and 998 gene genealogies for SCD and SID respectively. Prior distributions for each demographic parameter under each model are in Table 3, Supplementary Table S3 and S9. Model posterior probabilities were calculated by a weighted multinomial logistic regression[24] for which we retained the best 25,000 simulations. The demographic parameters within each model were estimated from the 5,000 simulations closest to the observed dataset using a local linear regression according to[22]. Analyses were performed in the R environment[20] with the library *abc*[25]. Posterior distributions of the estimated parameters for model CHG1 and FIM are shown in Supplementary Figure 8 and 9.

Cross-validation of the model selection was performed by randomly generating pseudo-observed datasets (*pods*) from prior distributions under each model. We simulated 1000 *pods* under each model and then we checked how many *pods* were correctly assigned to the true model with several thresholds of probability (from 0.95 to 0.50, see Supplementary Table S6). We note that COS and CHG1 are nested, which means that they can be difficult to distinguish under some parameter combinations (i.e., when the resize parameter is around 1). For this reason, we performed a cross-validation experiment of the model selection testing COS against CHG1. We plot the probability of correctly assigning a dataset as a function of the resize parameter: as expected the model selection procedure works fine and the two models cannot be distinguished exactly when the resize is around 1 (i.e., when CHG1 and COS are indeed the same), as suggested by Supplementary Figure S13.

The same procedure was used for the cross-validation of the parameter estimation. The coverage 95%, the scaled mean error (SME, calculated as in [26]) and scaled root mean square error (SRMSE, calculated as in [26]) were computed for each parameter of the model CHG1 and FIM for both datasets (Supplementary Table S7, Supplementary Fig. S10 and S11). SME and SRMSE were calculated on both the median and the mode of each estimated parameter.

A posterior predictive test[27] was carried out to test whether the data can be reproduced under a specific demographic model. We simulated 10,000 *pods* under each model using parameters from the ABC posterior distributions. For each *pods,* the number of polymorphic sites was calculated. The posterior distribution of the 10,000 numbers of polymorphic sites was then plotted together with the real value (in red). For all models, the real value was always within the 95% CI of the distribution and Bayesian p-values computed from the posterior distribution of the number of polymorphic sites showed that none of the four models in both datasets could be rejected (Supplementary Fig. S12). This is important as it highlights that none of our models was unrealistic or affected by major misspecification of prior distributions. We also performed 10,000 simulations of 14 independent STRs under the FIM model with parameters drawn from the posterior distribution of FIM (SID dataset). We used two mutation rates (0.0005 and 0.00001 per locus per generation) as in Vignaud et al. 2014. We calculated the expected *Fst* between two demes of the FIM model made by 15 diploid individuals each.

Supplementary References

1 Li, C., Hofreiter, M., Straube, N., Corrigan, S. & Naylor, G. J. Capturing protein-coding genes across highly divergent species. *Biotechniques* **54**, 321-326, (2013).

2 Naylor, G. J. P. *et al.* in *Biology of sharks and their relatives.* (eds J.C. Carrier, J.A. Musick, & M.R. Heithaus) 31-56 (CRC Press, 2012).

3 Naylor, G. J. P. *et al.* A DNA Sequence-Based Approach to the Identification of Shark and Ray Species and Its Implications for Global Elasmobranch Diversity and Parasitology. *B Am Mus Nat Hist*, 1-262, (2012).

4 Krueger, F. *Trim Galore!*, <http://www.bioinformatics.babraham.ac.uk/projects/trimgalore/> (2012).

5 Birol, I. *et al.* De novo transcriptome assembly with ABySS. *Bioinformatics* **25**, 2872-2877, (2009).

6 Robertson, G. *et al.* De novo assembly and analysis of RNA-seq data. *Nat Methods* **7**, 909-912, (2010).

7 Ebersberger, I., Strauss, S. & von Haeseler, A. HaMStR: profile hidden markov model based search for orthologs in ESTs. *Bmc Evolutionary Biology* **9**, 157, (2009).

8 Altschul, S. F. *et al.* Gapped BLAST and PSI-BLAST: a new generation of protein database search programs. *Nucleic Acids Res* **25**, 3389-3402, (1997).

9 Birney, E., Clamp, M. & Durbin, R. GeneWise and Genomewise. *Genome Res* **14**, 988-995, (2004).

10 Eddy, S. R. Accelerated Profile HMM Searches. *PLoS Comput Biol* **7**, e1002195, (2011).

11 Eddy, S. R. Profile hidden Markov models. *Bioinformatics* **14**, 755-763, (1998).

12 ea-utils: Command-line tools for processing biological sequencing data. (2011).

13 Li, H. & Durbin, R. Fast and accurate short read alignment with Burrows-Wheeler transform. *Bioinformatics* **25**, 1754-1760, (2009).

14 McKenna, A. *et al.* The Genome Analysis Toolkit: a MapReduce framework for analyzing next-generation DNA sequencing data. *Genome Res* **20**, 1297-1303, (2010).

15 Lessios, H. A. The Great American Schism: Divergence of Marine Organisms After the Rise of the Central American Isthmus. *Annu Rev Ecol Evol S* **39**, 63-91, (2008).

16 Drummond, A. J., Suchard, M. A., Xie, D. & Rambaut, A. Bayesian phylogenetics with BEAUti and the BEAST 1.7. *Mol Biol Evol* **29**, 1969-1973, (2012).
17 Hasegawa, M., Kishino, H. & Yano, T. Dating of the human-ape splitting by a molecular clock of mitochondrial DNA. *J Mol Evol* **22**, 160-174, (1985).
18 Rambaut, A., Suchard, M. A., Xie, D. & Drummond, A. J. *Tracer v1.6*, <http://beast.bio.ed.ac.uk/Tracer> (2014).
19 Mourier, J., Mills, S. C. & Planes, S. Population structure, spatial distribution and life-history traits of blacktip reef sharks Carcharhinus melanopterus. *J Fish Biol* **82**, 979-993, (2013).
20 R: A Language and Environment for Statistical Computing (R Foundation for Statistical Computing, 2014).
21 Nei, M. Analysis of gene diversity in subdivided populations. *Proc Natl Acad Sci U S A* **70**, 3321-3323, (1973).
22 Beaumont, M. A., Zhang, W. & Balding, D. J. Approximate Bayesian computation in population genetics. *Genetics* **162**, 2025-2035, (2002).
23 Excoffier, L., Dupanloup, I., Huerta-Sanchez, E., Sousa, V. C. & Foll, M. Robust demographic inference from genomic and SNP data. *PLoS Genet* **9**, e1003905, (2013).
24 Beaumont, M. A. in *Simulation, Genetics, and Human Prehistory* 135-154 (McDonald Institute for Archaeological Research, 2008).
25 Csillery, K., Francois, O. & Blum, M. G. B. abc: an R package for approximate Bayesian computation (ABC). *Methods Ecol Evol* **3**, 475-479, (2012).
26 Walther, B. A. & Moore, J. L. The concepts of bias, precision and accuracy, and their use in testing the performance of species richness estimators, with a literature review of estimator performance. *Ecography* **28**, 815-829, (2005).
27 Gelman, A., Carlin, J., Stern, H. & Rubin, D. *Bayesian Data Analysis*. (CRC Press, 2004).

Supplementary Tables

Supplementary Table S1: Summary of sampling locations. SCD: scatter dataset; SID: single deme dataset.

ID	Sampling location	Region	Country	Dataset
261	Anda	Pangasinan	Philippines	SCD
1175	Bangsaray		Thailand	SCD
3599	Labuan	Sabah	Malaysia	SCD
4223	Kota Baru		Indonesia	SCD
4617	Singkawang	South Kalimantan	Indonesia	SCD
4814	Tanjung Batu	West Kalimantan	Indonesia	SCD
9718	Daymanyat Island		Oman	SCD
5533	Weipa	Queensland	Australia	SCD/SID
5134	Dundee Beach	Northern Territory	Australia	SCD/SID
5135	Dundee Beach	Northern Territory	Australia	SID
5139	Dundee Beach	Northern Territory	Australia	SID
5146	Dundee Beach	Northern Territory	Australia	SID
5521	Weipa	Queensland	Australia	SID
5522	Weipa	Queensland	Australia	SID
5534	Weipa	Queensland	Australia	SID
5571	Weipa	Queensland	Australia	SID
1269	Dundee Beach	Northern Territory	Australia	SID
5133	Dundee Beach	Northern Territory	Australia	SID

Supplementary Table S2: Summary of the filtering steps for both datasets: "scatter" (SCD) and "single deme" (SID). MQ: mapping quality; BQ: base quality; DP: depth of coverage; SP: strand bias; NA: missing data.

Full dataset	No. of sites	No. of reference sites	No. of variant sites
MQ=0, BQ=20	1,292,003	1,282,374	9629
MQ=30, BQ=20	1,268,572	1,259,257	9315

	No. of genotypes	No. of reference genotypes	No. of variant genotypes
MQ=30, BQ=20	36,788,588	36,518,453	270,135
MQ=30, BQ=20, DP≥6	22,057,360	21,888,471	168,889
MQ=30, BQ=20, DP≥6, SP≤13	22,056,840	21,888,320	168,520

Samples defining the "scatter" (SCD) and "single deme" (SID) datasets

	SCD	SID
MQ=30, BQ=20, DP≥6, SP≤13, NA=0	610,274	640,402
MQ=30, BQ=20, DP≥6, SP≤13, NA=0, no triallelic sites	610,254	640,493
MQ=30, BQ=20, DP≥6, SP≤13, NA=0, no triallelic sites, no regions with excess of SNPs	606,647	632,160
Regions retained	995/1077	998/1077
Total number of SNPs	2605	1946
Total number of reference sites	604,042	630,214

SNP density (kb)	SCD	SID
Region	4.3	3.1
Exon	2.7	2.0
Introns (5′+3′)	5.3	3.8
Intron 5′	5.5	3.8
Intron 3′	5.7	3.8

Supplementary Table S3: parameter estimation under model CHG1. N_{mod}: modern effective population size; N_{anc}: ancestral effective population size; Resize: calculated as N_{anc}/N_{mod}; T_c: time of the demographic change (in generations); SCD: scatter dataset (18 chromosomes); SID: single deme dataset (22 chromosomes).

Model CHG1	Median	Mode	0.025[a]	0.975[a]	Prior[b]
**SCD**					
N_{mod}	106,706	101,305	88,523	158,512	U: 1000-300,000
N_{anc}	29,697	37,179	3075	58,241	U: 1000-300,000
**Resize**	0.267	0.333	0.031	0.538	
T_c	82,553	92,126	27,206	147,548	U: 100-200,000
**SID**					
N_{mod}	61,306	57,276	50,970	99,134	U: 1000-300,000
N_{anc}	28,210	13,947	1844	259,262	U: 1000-300,000
**Resize**	0.480	0.263	0.028	2.56	
T_c	79,687	63,319	10,283	175,152	U: 100-200,000

[a]Upper and lower limits of the 95% credible interval about the estimated mode.
[b]U, uniform probability, in the range of the two values.

Supplementary Table S4: models posterior probability calculated as in [24] using the closest 25,000 simulations. SCD: scatter dataset; SID: single deme dataset.

Dataset	CHG1	CHG2
SCD	0.50	0.50
SID	0.51	0.49

Supplementary Table S5: Gst distance matrix[21] across all populations.

Gst matrix	Philippines	Thailand	Northern territory	Malaysia	South Kalimantan	West Kalimantan	East Kalimantan	Queensland	Oman
Philippines	-								
Thailand	0.34	-							
Northern territory	0.29	0.31	-						
Malaysia	0.33	0.35	0.32	-					
South Kalimantan	0.31	0.33	0.27	0.33	-				
West Kalimantan	0.33	0.31	0.27	0.33	0.30	-			
East Kalimantan	0.32	0.35	0.29	0.34	0.32	0.32	-		
Queensland	0.30	0.32	0.05	0.32	0.28	0.28	0.29	-	
Oman	0.55	0.56	0.45	0.57	0.52	0.51	0.53	0.45	-

Supplementary Table S6: Cross-validation of the model selection for both sampling schemes. We simulated 1000 pseudo-observed data sets (*pods*) under each model and then we checked how many *pods* were correctly assigned to the model used to simulate them with several thresholds of probability (from 0.95 to 0.50). SCD: scatter dataset; SID: single deme dataset.

SCD	COS						CHG1						FIM					
**Model**	**0.95**	**0.90**	**0.80**	**0.70**	**0.60**	**0.50**	**0.95**	**0.90**	**0.80**	**0.70**	**0.60**	**0.50**	**0.95**	**0.90**	**0.80**	**0.70**	**0.60**	**0.50**
**COS**	0	0	0	98	293	528	0	0	0	1	4	70	0	0	0	0	0	0
**CHG1**	0	0	0	24	129	248	29	53	110	156	204	301	0	0	9	68	142	191
**FIM**	0	0	0	0	0	2	0	0	8	16	26	59	13	103	340	511	635	721

SID	COS						CHG1						FIM					
**Model**	**0.95**	**0.90**	**0.80**	**0.70**	**0.60**	**0.50**	**0.95**	**0.90**	**0.80**	**0.70**	**0.60**	**0.50**	**0.95**	**0.90**	**0.80**	**0.70**	**0.60**	**0.50**
**COS**	0	0	0	136	309	544	0	0	0	0	3	87	0	0	0	0	1	6
**CHG1**	0	0	0	22	120	234	59	93	134	179	248	340	0	0	5	85	154	197
**FIM**	0	0	0	0	0	3	0	0	3	9	27	50	6	78	260	491	647	716

Supplementary Table S7: Cross-validation of the parameter estimation (mode and median) for both sampling schemes. SME: scaled mean error calculated as in [26], SRMSE: scaled root mean squared error calculated as in [26]. N_{mod}: modern effective population size; N_{anc}: ancestral effective population size; Resize: calculated as N_{anc}/N_{mod}; T_c: time of the demographic change (in generations); T_i: time of the onset of the island (in generations); *Nm*: product of the effective population size *N* and the migration rate *m* for each deme; SCD: scatter dataset (18 chromosomes); SID: single deme dataset (22 chromosomes).

SCD

CHG1

	N_{mod}	N_{anc}	T_c
Coverage 95%	0.967	0.972	0.947
SME mode	0.020	0.140	1.154
SRMSE mode	0.491	1.321	7.770
SME median	0.032	0.280	1.536
SRMSE median	0.481	1.868	10.919

FIM

	N_{anc}	T_i	*Nm*
Coverage 95%	0.96	0.97	0.96
SME mode	1.38	0.48	-0.02
SRMSE mode	20.31	9.76	0.32
SME median	1.26	0.76	0.03
SRMSE median	17.35	7.20	0.44

SID

CHG1

	N_{mod}	N_{anc}	T_c
Coverage 95%	0.963	0.954	0.965
SME mode	-0.001	0.253	1.090
SRMSE mode	0.153	2.376	11.096
SME median	0.012	0.440	1.253
SRMSE median	0.144	2.625	9.490

FIM

	N_{anc}	T_i	*Nm*
Coverage 95%	0.95	0.97	0.96
SME mode	0.88	0.68	-0.01
SRMSE mode	5.72	7.47	0.67
SME median	0.93	2.17	0.04
SRMSE median	5.36	29.97	0.91

Supplementary Table S8: Software tools and parameters used in data analysis.

Function	Tool	Package	Parameters
Quality control	FastQC		N/A
Adapter trimming	Fastq-mcf		N/A
Map reads	BWA v0.7.10		aln –q15 reference.fa R1.fq R1.sai<br>aln –q15 reference.fa R2.fq R2.sai<br>sampe reference.fa R1.sai R2.sai R1.fq R2.fq > R1_R2.sam
Duplicate marking	MarkDuplicates	Picard v1.129	N/A
Local realignment	RealignerTargetCreator	GATK v3.3-0	-R reference.fa
	IndelRealigner		-R reference.fa -targetIntervals file_made_by_RealignerTargetCreator
Raw sequence depth	DepthOfCoverage	GATK v3.3-0	N/A
SNP calling	mpileup	SAMtools v1.1	-AI -Q 20 -q 30 --output-tags DP,DV,DPR,INFO/DPR,SP -g -O -s –f reference.fa –b list_bam_files
	bcftools call		-m -
Filtering	Perl and R scripts		Strand bias (SP≤13)<br>Depth of coverage (DP≥6)<br>Remove triallelic sites<br>Remove any missing data

Supplementary Table S9: parameter estimation under the constant-size model (COS). N: effective population size; SCD: scatter dataset (18 chromosomes); SID: single deme dataset (22 chromosomes)

Model COS	Median	Mode	0.025[a]	0.975[a]	Prior[b]
**SCD**					
**N**	76,617	76,648	72,291	81,129	U: 1000-300,000
**SID**					
**N**	51,177	50,939	48,231	54,190	U: 1000-300,000

Supplementary Figure S1: Length distribution of regions included in SID and SCD.
a) length distribution of 998 regions in SID; b) length distribution of 995 regions in SCD.

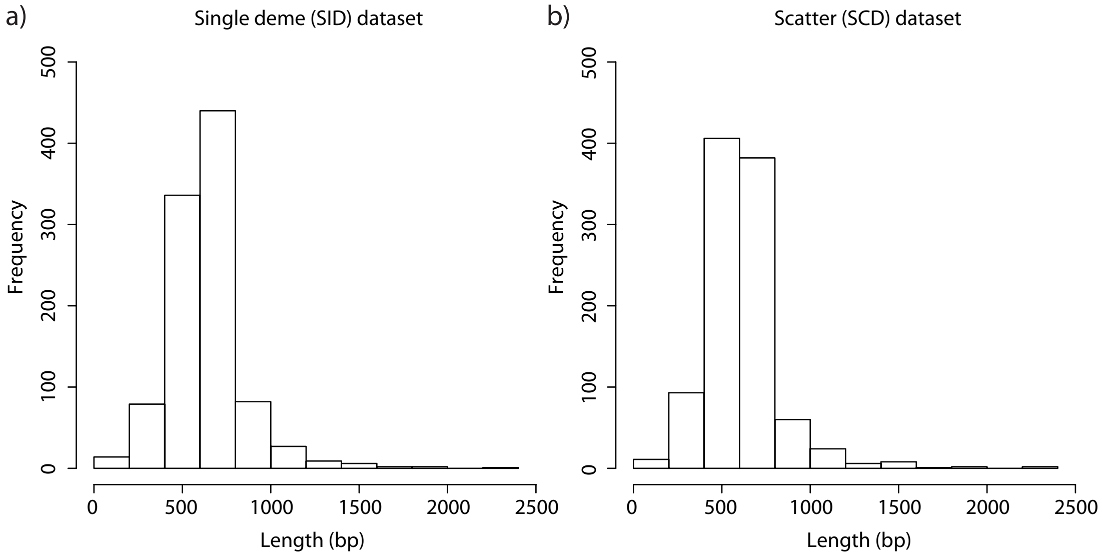

Supplementary Figure S2: Principal Component analysis.
a) and b) 18 samples; c) and d) 17 samples, excluding those from Oman.

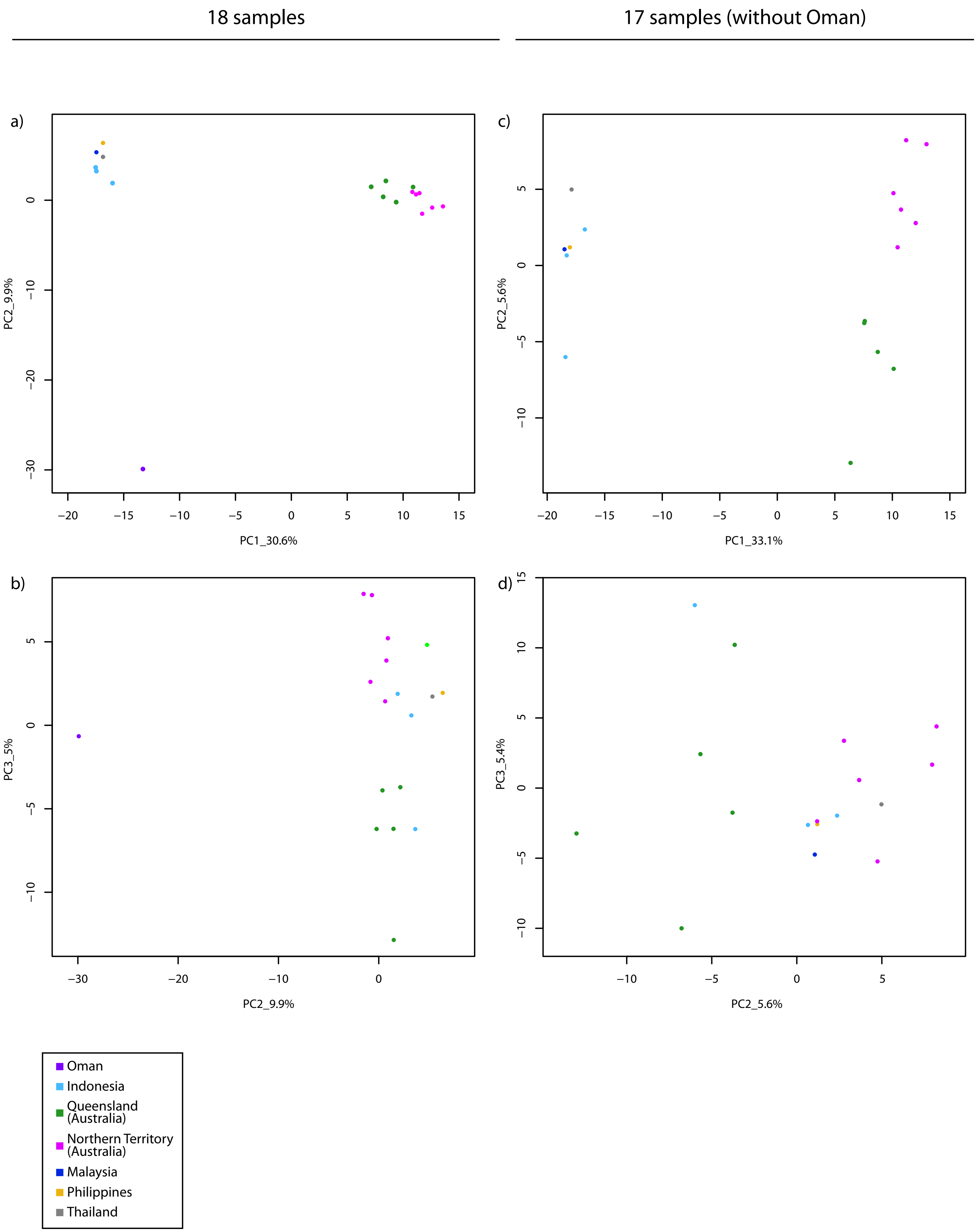

Supplementary Figure S3: Skyline reconstruction of the effective population size through time under CHG2. a) single deme (SID); b) scatter (SCD). Both skylines were reconstructed up to the mode of the mean TMRCA across loci. Median values are shown (darker lines) with the 95% high posterior density interval (lighter lines).

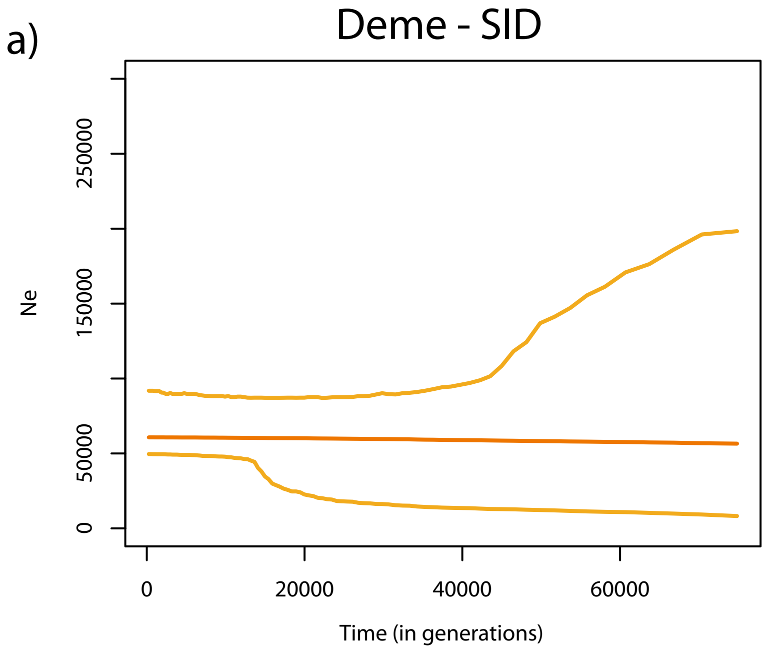

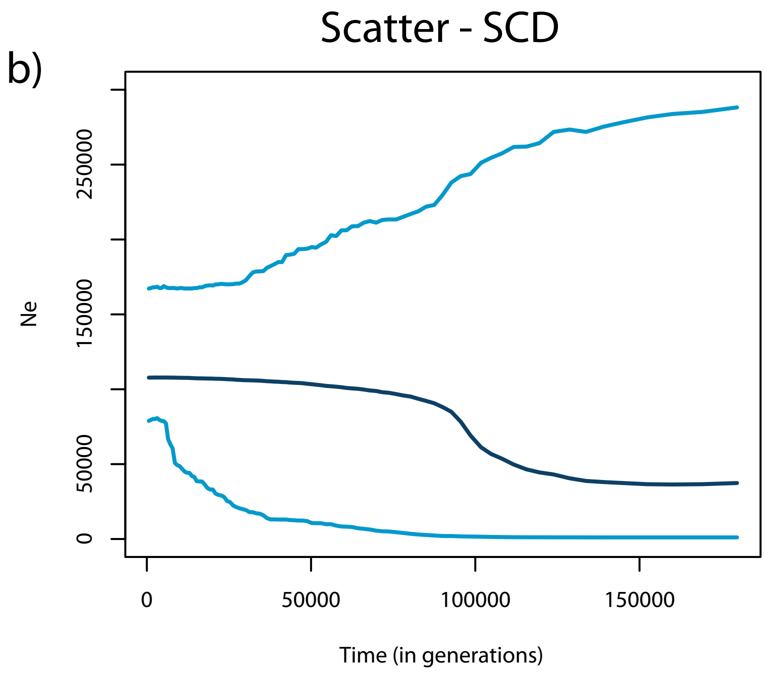

Supplementary Figure S4: Demographic models tested for the detection of a recent bottleneck.
a) CHG1-BOTT, demographic-change model (one demographic change) with a bottleneck; b) FIM-BOTT, non-equilibrium finite island model with bottleneck. N_{mod}: modern effective population size; N_{anc}: ancestral effective population size; T_c: time of the demographic change (in generations); T_i: time of the onset of the island (in generations); T_{bott}: time when an instantaneous decrease in connectivity occurred.

a) Model CHG1-BOTT

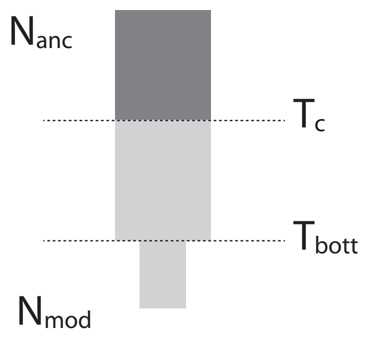

b) Model FIM-BOTT

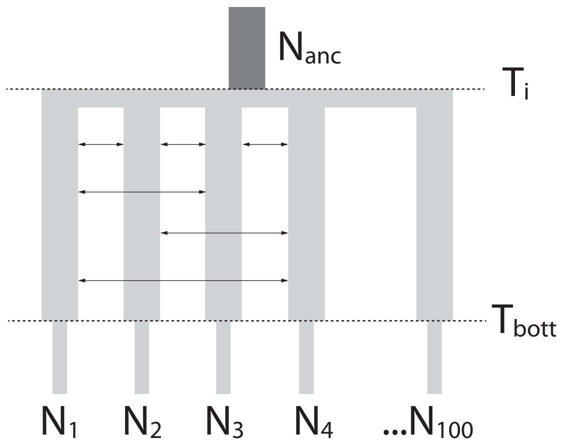

Supplementary Figure S5: Average skyline reconstruction under model CHG2 of pseudo-observed data sets (pods) simulated with model FIM and FIM-BOTT in a metapopulation (for SCD and SID) and in an unstructured population (model CHG1 and CHG1-BOTT).

Data were simulated with I_{bott}=100 at: a) T_{bott}=10 generations; b) T_{bott}=50 generations; c) T_{bott}=100 generations; d) T_{bott}=200 generations and d) T_{bott}=500 generations before present. An average skyline reconstruction is shown across 1000 simulations. Solid lines: scenario with bottleneck (model FIM-BOTT and CHG1-BOTT); dashed line: scenario without bottleneck (model FIM and CHG1). Median values are shown (darker lines) with the 95% high posterior density interval (lighter lines).

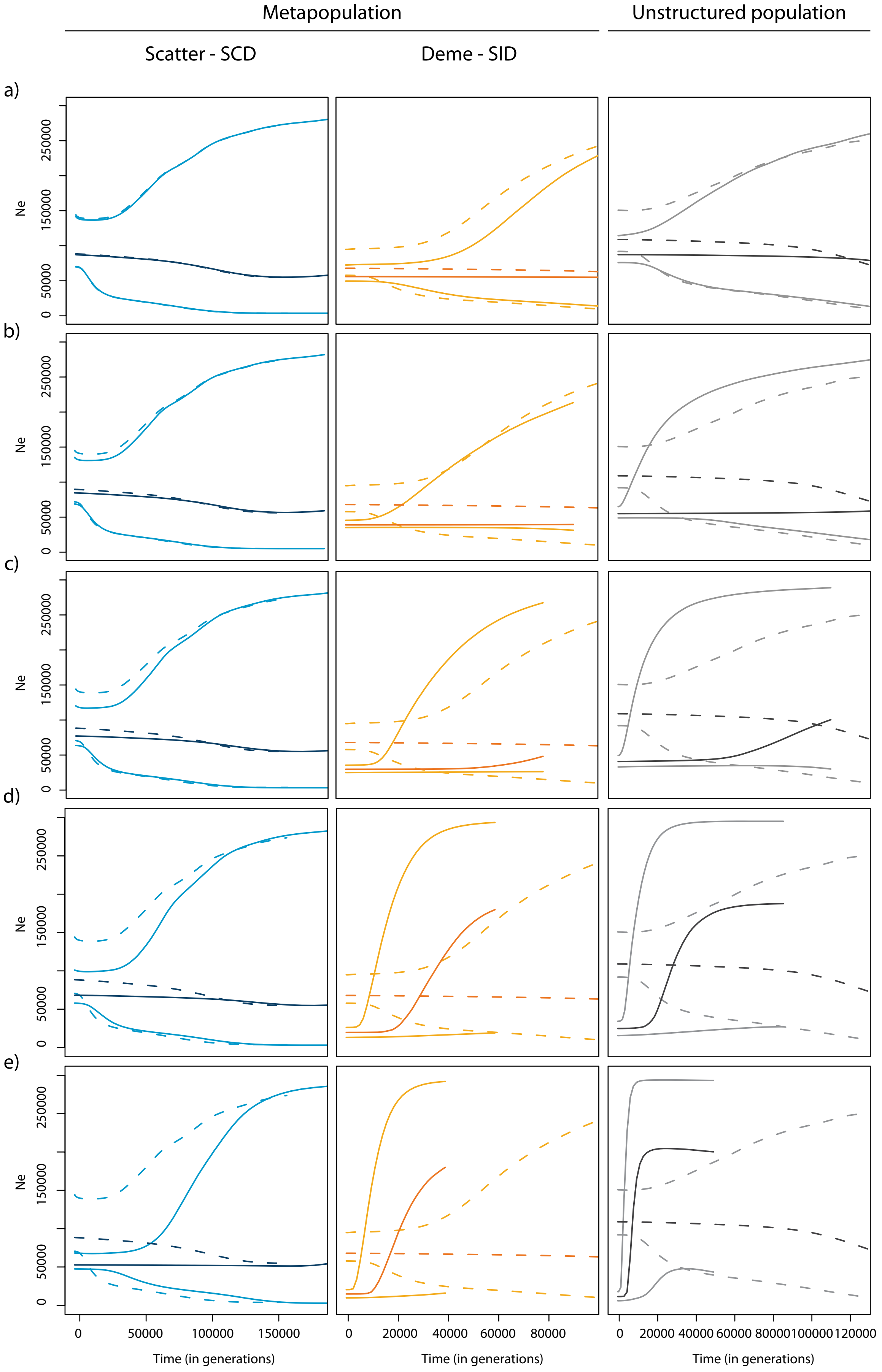

Supplementary Figure S6: Average skyline reconstruction under model CHG2 of pseudo-observed data sets (pods) simulated with model FIM and FIM-BOTT in a metapopulation (for SCD and SID) and in an unstructured population (model CHG1 and CHG1-BOTT).

Data were simulated with I_{bott}=1000 at: a) T_{bott}=10 generations; b) T_{bott}=50 generations; c) T_{bott}=100 generations; d) T_{bott}=200 generations and d) T_{bott}=500 generations before present. An average skyline reconstruction is shown across 1000 simulations. Solid lines: scenario with bottleneck (model FIM-BOTT and CHG1-BOTT); dashed line: scenario without bottleneck (model FIM and CHG1). Median values are shown (darker lines) with the 95% high posterior density interval (lighter lines).

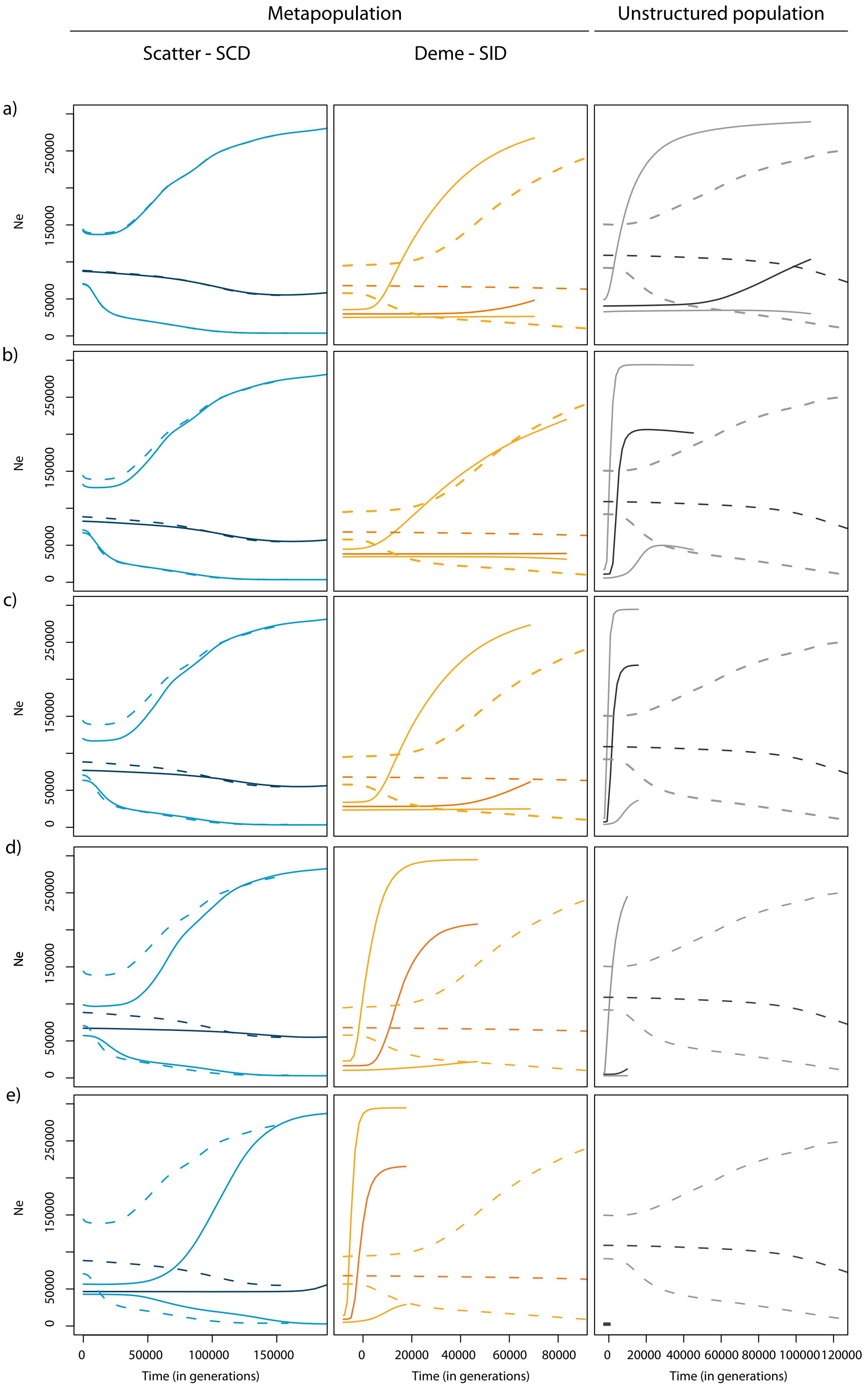

Supplementary Figure S7: Distribution of the median of *Nm* estimated under model FIM-BOTT from pseudo-observed data sets (*pods*) generated with FIM-BOTT and FIM.

Data were simulated for both sampling schemes with I_{bott}=1000 and various T_{bott} (10, 50, 100, 200, 500 and 1000 generations ago). Dotted lines represent the value of *Nm* used to simulate pods under FIM model.

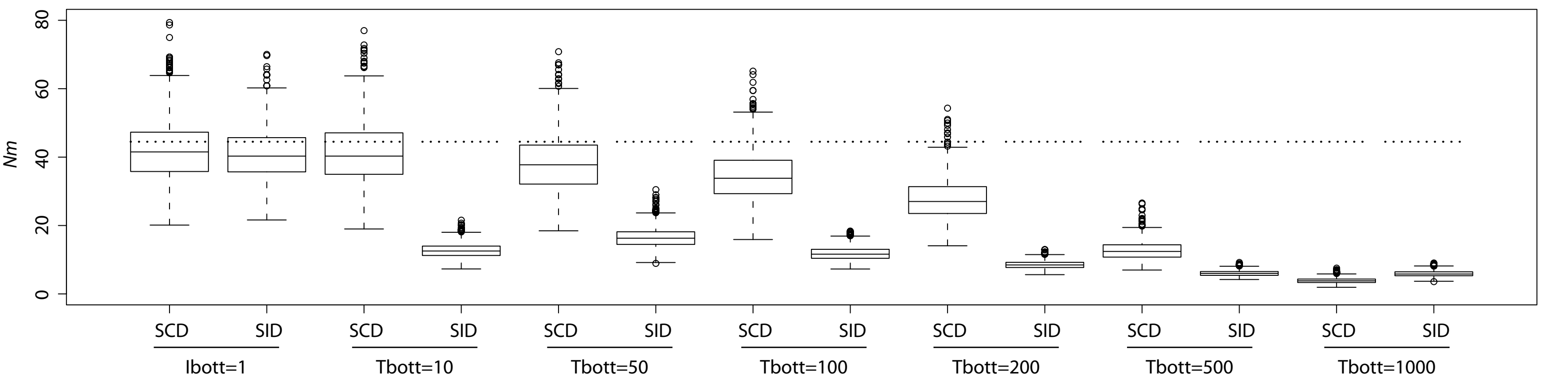

Supplementary Figure S8: Posterior distributions for the parameters estimated under model CHG1. The time of the demographic change (T_c) and both the modern (N_{mod}) and ancestral (N_{anc}) effective population sizes are shown for both sampling schemes (SCD and SID). Black line: prior distribution; red line: posterior distribution calculated using a local linear regression according to 22.

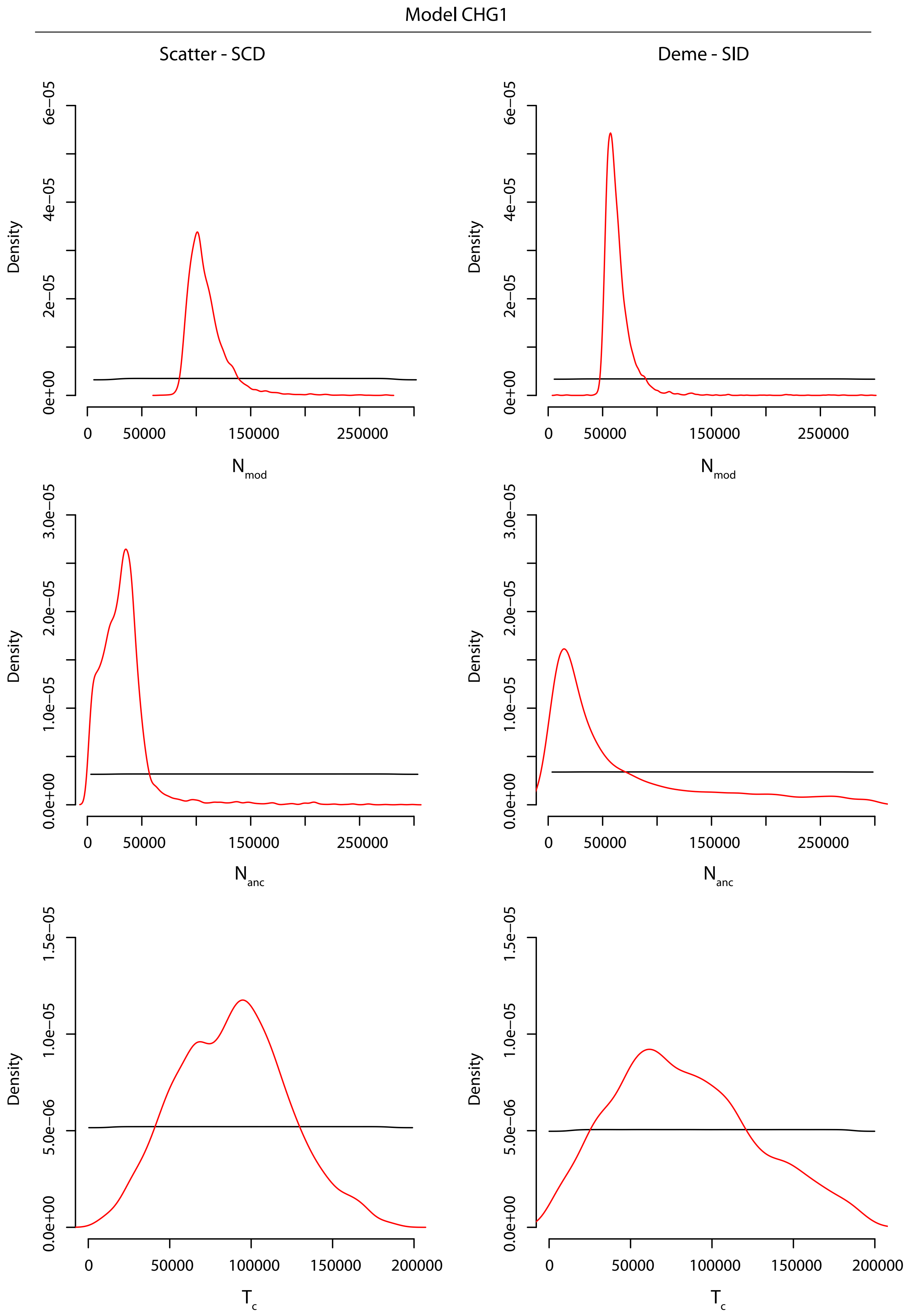

Supplementary Figure S9: Posterior distributions for the parameters estimated under model FIM. The effective population size of the ancestral deme (N_{anc}), the time of the onset of the island (T_i) and Nm are shown for SCD, SID and pooled samples (SCD+SID without shared samples). Black line: prior distribution; red line: posterior distribution calculated using a local linear regression according to 22.

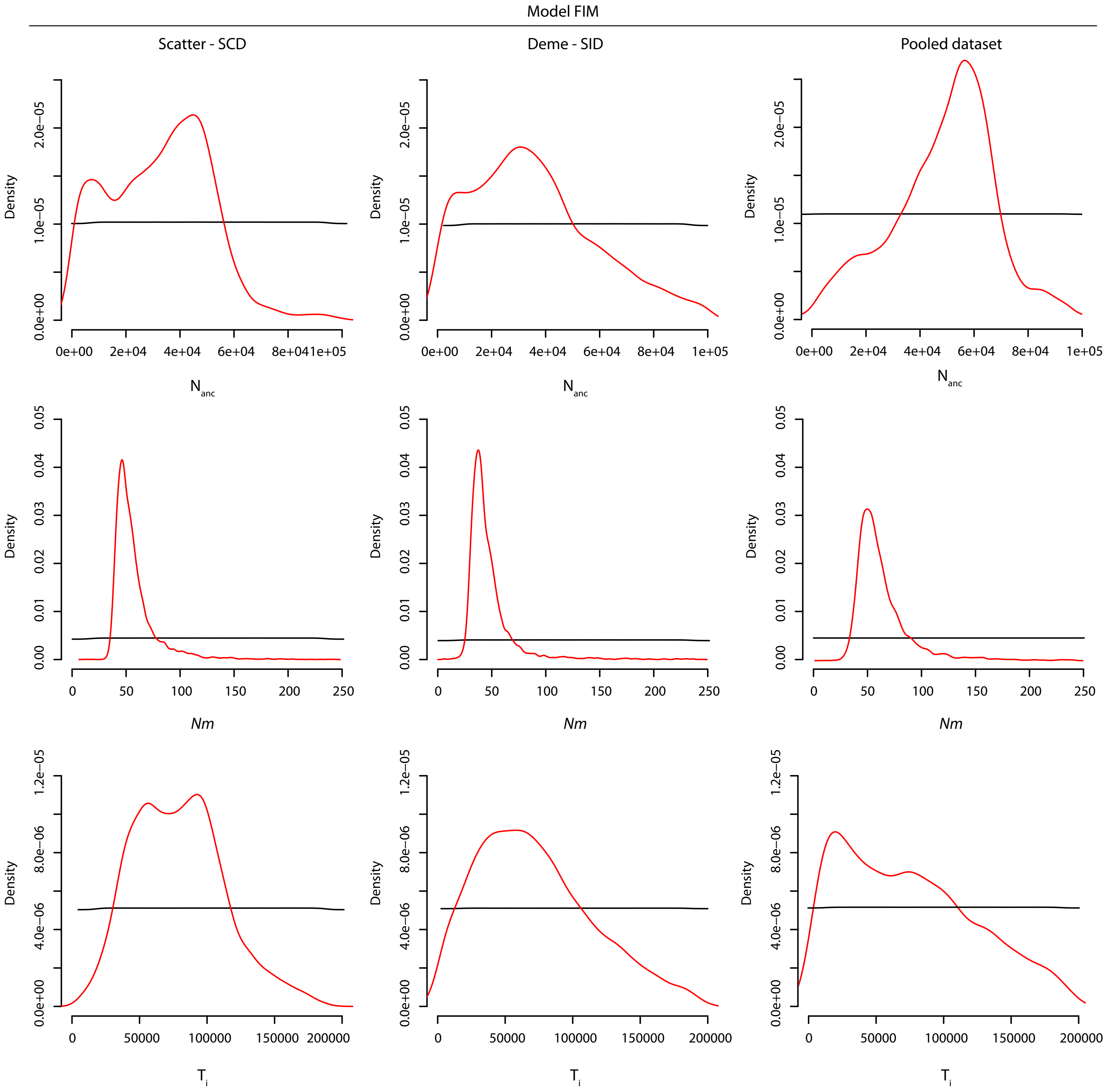

Supplementary Figure S10: Cross-validation of the parameter estimation for model FIM.
The effective population size of the ancestral deme (N_{anc}), the time of the onset of the island (T_i) and *Nm* are shown for both sampling schemes (SCD and SID).

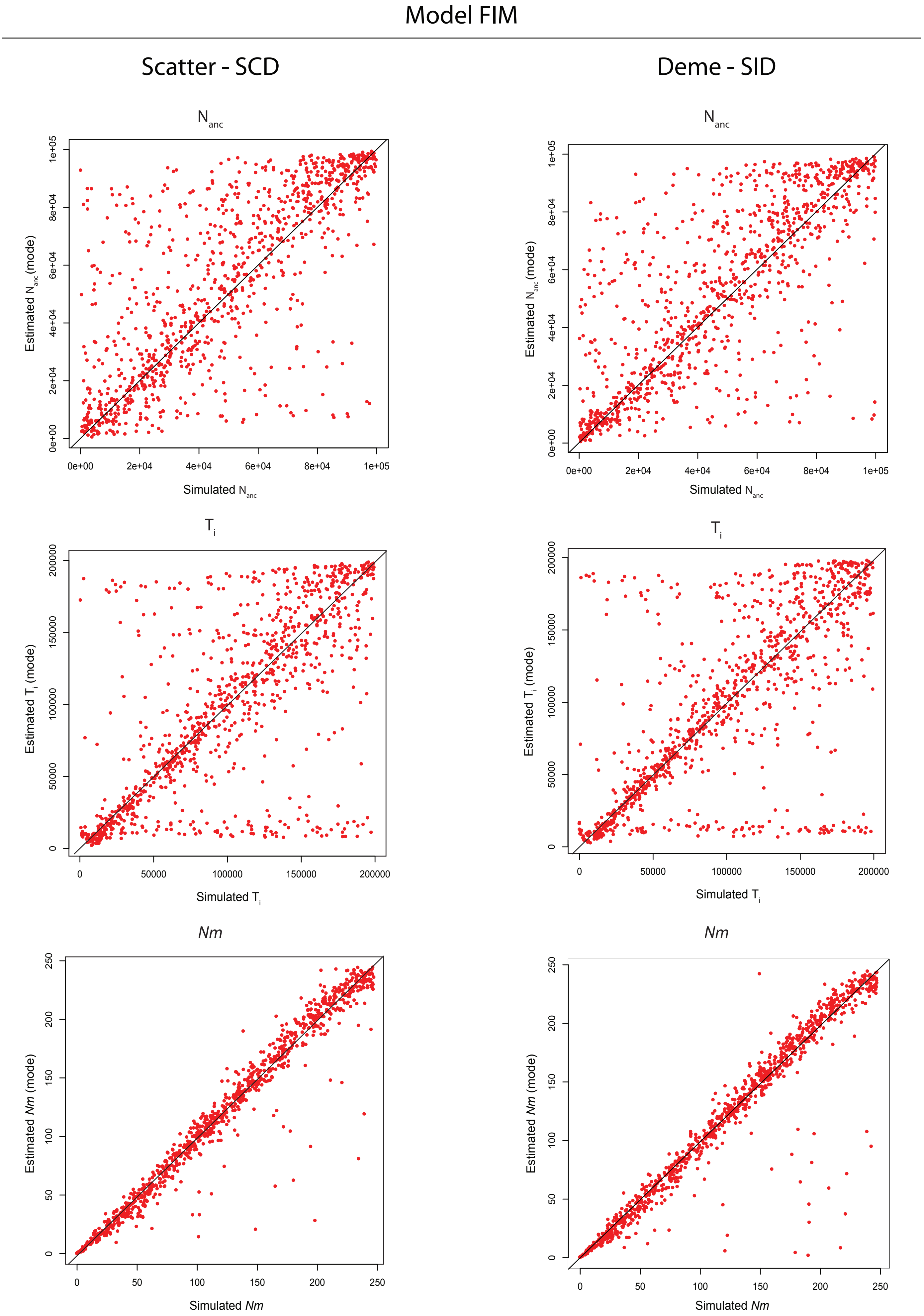

Supplementary Figure S11: Cross-validation of the parameter estimation for model CHG1. The time of the demographic change (T_c) and both the modern (N_{mod}) and ancestral (N_{anc}) effective population sizes are shown for both sampling schemes (SCD and SID).

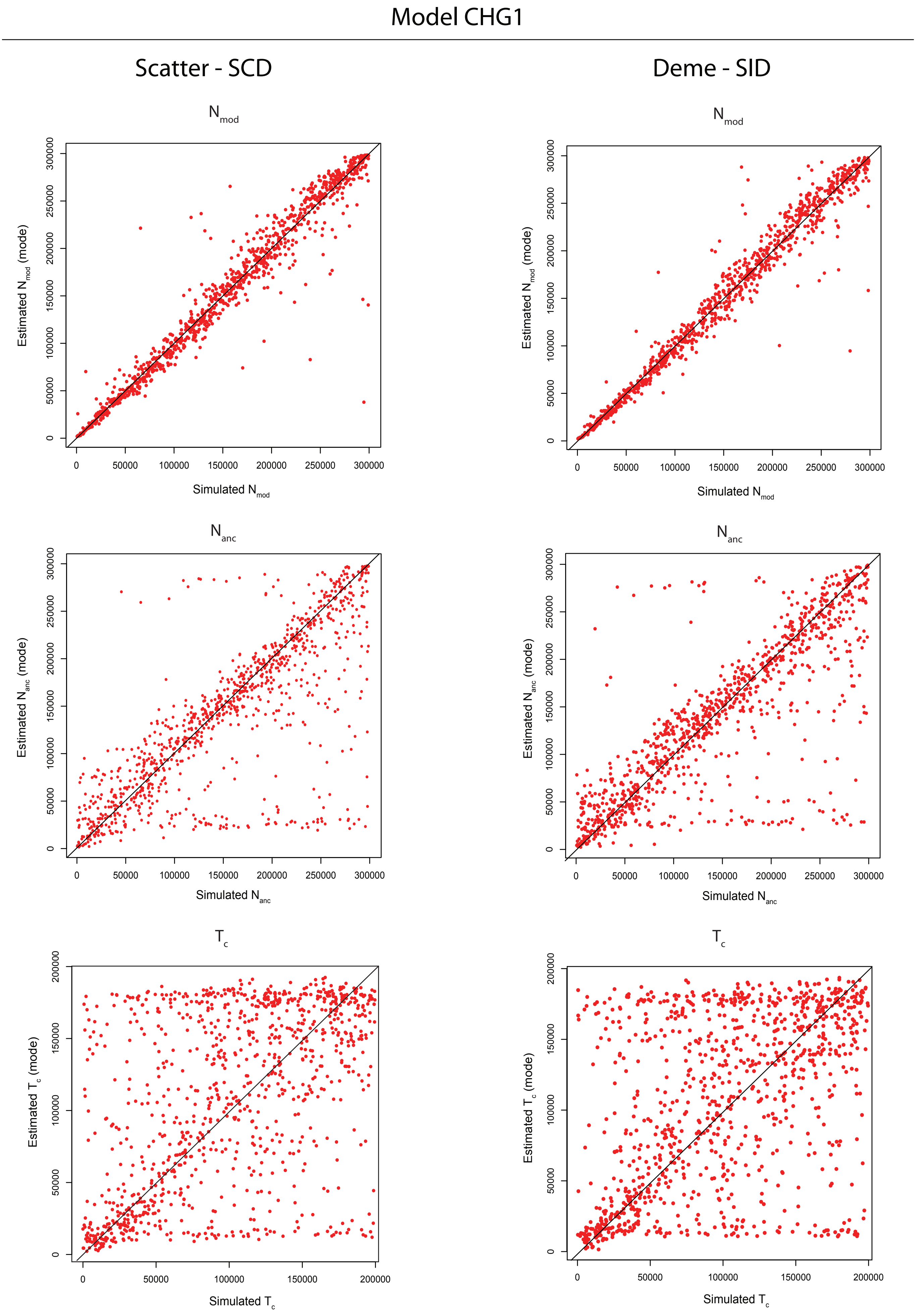

Supplementary Figure S12: Posterior predictive test for COS, CHG1 and FIM for both sampling schemes (SCD and SID).
In red the real number of SNPs is shown.

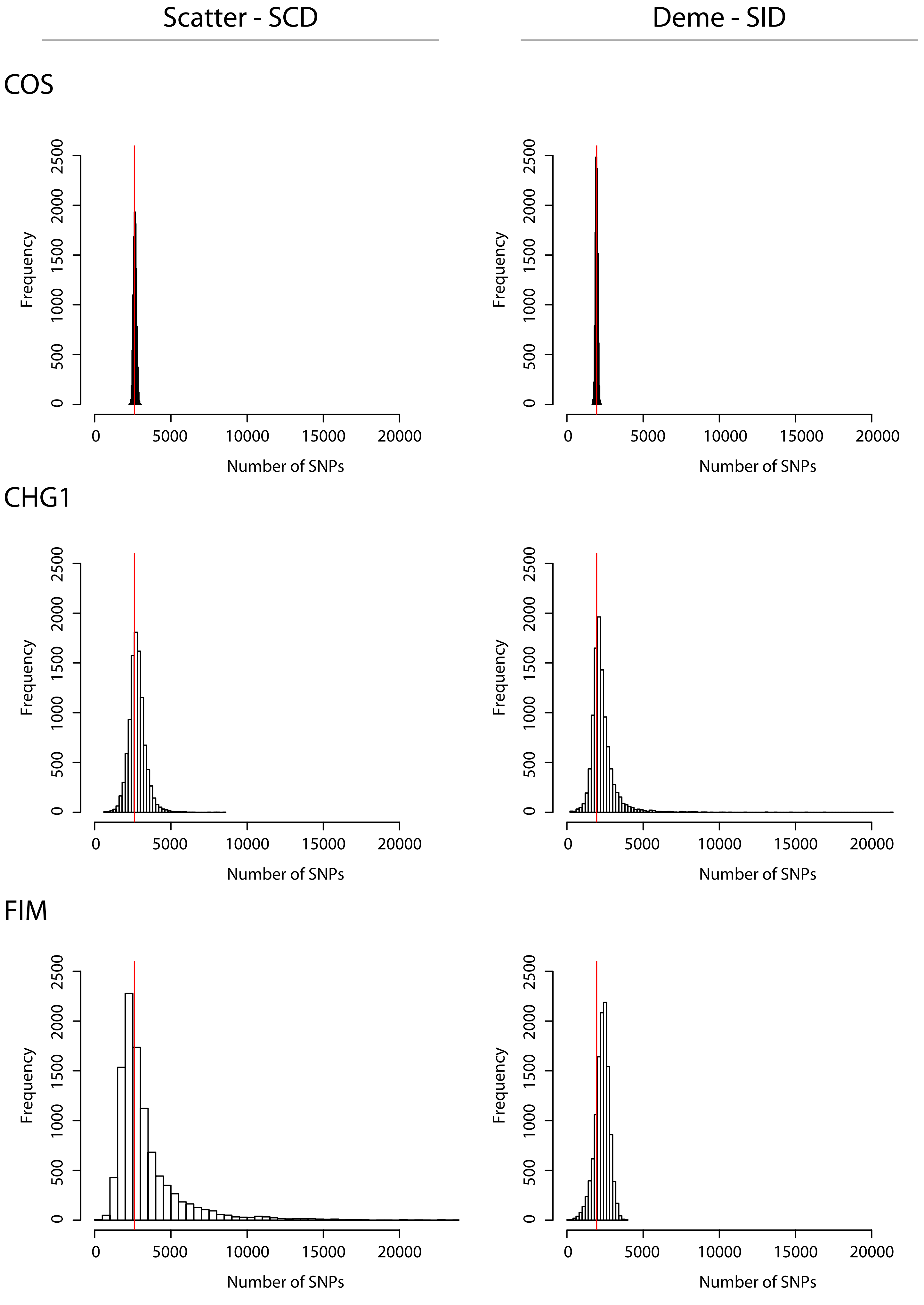

Supplementary Figure S13: Power of discriminating model CHG1 based on resize (N_{anc}/N_{mod}) values.
For this analysis 10,000 *pods* were generated and the probability of model CHG1 was plotted against its corresponding value of resize. The power to distinguish model CHG1 and COS decreases when resize = 1 (model CHG1 and COS are the same).

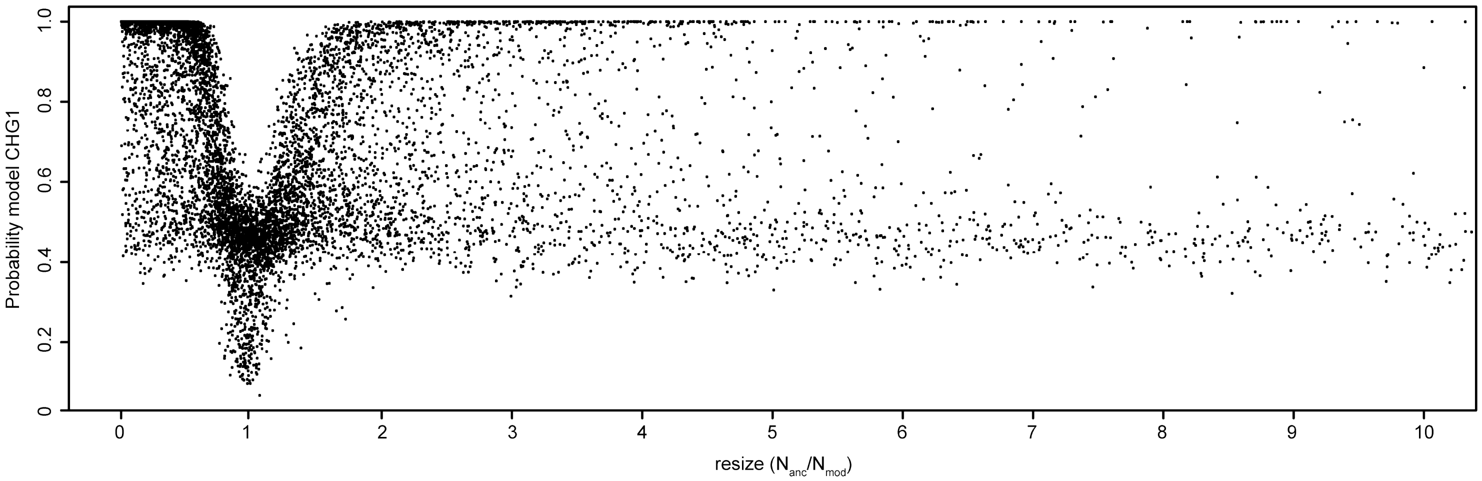

Supplement: Supplementary Information [file srep33753-s1.pdf]
